# Draft Genome Sequence of *Mycobacterium obuense* Strain UC1, Isolated from Patient Sputum

**DOI:** 10.1128/genomeA.00612-15

**Published:** 2015-06-11

**Authors:** Alexander L. Greninger, Gail Cunningham, Elaine D. Hsu, Joanna M. Yu, Charles Y. Chiu, Steve Miller

**Affiliations:** Department of Laboratory Medicine, UCSF, San Francisco, California, USA

## Abstract

We report the draft genome sequence of *Mycobacterium obuense* strain UC1 from a patient sputum sample. This is the first draft genome sequence of *Mycobacterium obuense*, a rapidly growing scotochromogenic mycobacterium.

## GENOME ANNOUNCEMENT

*Mycobacterium obuense* is a rapidly growing scotochromogenic member of the genus *Mycobacterium* that has been previously isolated from soil and the sputum of a patient with pulmonary disease (1). The unique biochemical properties of *M. obuense* that resulted in it being classified as a species include its ability to degrade *p*-aminosalicylate and salicylate into black products. Smooth colonies of this species have been reported to be mobile, along with several other environmental mycobacteria (2). A heat-killed suspension of *M. obuense* has been investigated as a potential treatment for melanoma due to its stimulation of antitumor T cell response (3, 4).

Rapidly growing mycobacteria constitute a commonly isolated population of acid-fast bacilli in the clinical microbiology lab of varying clinical importance (5). Their speciation via molecular methods is becoming increasingly important given the expanding numbers of immunosuppressed individuals in which they can be pathogenic. We sequenced the first draft genome of *Mycobacterium obuense* in order to enhance molecular methods of detection and to understand mycobacterial genetic diversity.

The patient had a history of non–small cell lung cancer and presented with patchy lung infiltrates. A sputum culture for acid-fast bacilli yielded a rapidly growing scotochromogen identified by high-performance liquid chromatography as *M. obuense*/*M. aurum*. DNA from isolated colonies of strain UC1 was extracted using the Qiagen EZ1 DNA tissue kit and paired-end libraries were prepared using the Nextera XT DNA library kit followed by sequencing on the Illumina MiSeq. Sequences were adapter and quality (Q20) trimmed using cutadapt, *de novo* assembled using SPAdes version 3.5, metagenomically screened with SURPI, and annotated with Prokka version 1.1 (6–9). A total of 10,848,516 paired-end reads of average length 111.7 nucleotides were recovered after trimming. *De novo* assembly yielded 249 contigs >500 nucleotides for a total assembly size of 6,382,135 bp with an *N*_50_ of 55,467 bp, an average coverage of 148×, and a total of 6,193 coding sequences. Contiguity was most likely disrupted by high GC content (68%), and several high-copy-number integrases and transposases longer than the sequence read length. Of note, three contigs >18 kb each demonstrated 4× higher coverage than other sequences and aligned to plasmids present in *M. abscessus* strains with approximately 80% identity, suggesting that the UC1 strain has one or more plasmids.

Pairwise alignment of available sequenced genes from *Mycobacterium obuense* type strain CIP 106803 (16S, *hsp65*, *gyrB*, *rpoB*, *sod*) with strain UC1 revealed 99 to 100% identity, confirming the identify as *Mycobacterium obuense*. The closest related complete genome to *M. obuense* UC1 was *Mycobacterium chubuense* NBB4 (81.3% identity), while the closest NCBI WGS database entry was *Mycobacterium rufum* JS14 (92.2% identity). Analysis by the Comprehensive Antibiotic Resistant Database identified putative resistance genes *marA* (55% amino acid identity to *Frankia* spp.), aminoglycoside resistance protein/kinase (62% amino acid identity to *M. rufum* JS14), two metallo-beta-lactamases (86% and 89% amino acid identity to *M. rufum* JS14), *blaF* beta-lactamase (79% amino acid to *M. rufum* JS14), and beta-lactamase (43% amino acid to *Gordonia* spp.) (10, 11).

### Nucleotide sequence accession numbers.

This whole-genome shotgun project has been deposited at DDBJ/ENA/GenBank under the accession number LAUZ00000000. The version described in this paper is the second version, LAUZ02000000.
